# Behavioural and neurochemical mechanisms underpinning the feeding-suppressive effect of GLP-1/CCK combinatorial therapy

**DOI:** 10.1016/j.molmet.2020.101118

**Published:** 2020-11-19

**Authors:** Emma Roth, Simon Benoit, Baptiste Quentin, Brian Lam, Sarah Will, Marcella Ma, Nick Heeley, Tamana Darwish, Yashaswi Shrestha, Fiona Gribble, Frank Reimann, Irina Pshenichnaya, Giles Yeo, David J. Baker, James L. Trevaskis, Clemence Blouet

**Affiliations:** 1 MRC Metabolic Diseases Unit, University of Cambridge Metabolic Research Laboratories, WT-MRC Institute of Metabolic Science, University of Cambridge, Cambridge CB2 OQQ, UK; 2 Cardiovascular, Renal, and Metabolic Diseases, MedImmune LLC, Gaithersburg, MD 20878, USA; 3 Early Oncology, Translational Medicine, MedImmune LLC, Gaithersburg, MD 20878, USA; 4 Wellcome-MRC Cambridge Stem Cell Institute, Cambridge, UK; 5 Cardiovascular, Renal and Metabolic Diseases, MedImmune Ltd., Cambridge, UK

**Keywords:** Obesity, Satiety, Hunger, Nucleus of the solitary tract, GLP-1, CCK, Combinatorial therapy

## Abstract

**Objectives:**

Combinatorial therapies are under intense investigation to develop more efficient anti-obesity drugs; however, little is known about how they act in the brain to produce enhanced anorexia and weight loss. The goal of this study was to identify the brain sites and neuronal populations engaged during the co-administration of GLP-1R and CCK1R agonists, an efficient combination therapy in obese rodents.

**Methods:**

We measured acute and long-term feeding and body weight responses and neuronal activation patterns throughout the neuraxis and in specific neuronal subsets in response to GLP-1R and CCK1R agonists administered alone or in combination in lean and high-fat diet fed mice. We used PhosphoTRAP to obtain unbiased molecular markers for neuronal populations selectively activated by the combination of the two agonists.

**Results:**

The initial anorectic response to GLP-1R and CCK1R co-agonism was mediated by a reduction in meal size, but over a few hours, a reduction in meal number accounted for the sustained feeding suppressive effects. The nucleus of the solitary tract (NTS) is one of the few brain sites where GLP-1R and CCK1R signalling interact to produce enhanced neuronal activation. None of the previously categorised NTS neuronal subpopulations relevant to feeding behaviour were implicated in this increased activation. However, we identified NTS/AP *Calcrl*^+^ neurons as treatment targets.

**Conclusions:**

Collectively, these studies indicated that circuit-level integration of GLP-1R and CCK1R co-agonism in discrete brain nuclei including the NTS produces enhanced rapid and sustained appetite suppression and weight loss.

## Highlights

• GLP-1R/CCK1R co-agonism produces greater than additive feeding and weight-suppressive effects.
• GLP-1R/CCK1R co-agonism activates a greater than additive number of neurons in only a few brain areas, including the NTS.
• Previously categorised NTS and ARH populations are not increasingly activated in response to the combinatorial treatment.
• NTS/AP *Calcrl*^*+*^ neurons are activated by GLP-1R and CCK1R co-agonism but not in response to mono-agonism.
• AC187 blunted the feeding-suppressive effect of GLP-1R and CCK1R co-agonism.

## Abbreviations

*Adcyap1r1*: adenylate cyclase activating polypeptide 1 receptor type 1
AGRP: agouti-related peptide
AP: area postrema
ARH: arcuate nucleus of the hypothalamus
*Calcrl*: calcitonin receptor-like receptor gene
CCK: cholecystokinin
CCK1R: cholecystokinin receptor type A
CCK2R: cholecystokinin receptor type B
CeA: central amygdala
CLR: calcitonin receptor-like receptor
CT: calcitonin receptor
dER: differential enrichment ratio
DLPBN: dorsolateral parabrachial nucleus
DMH: dorsomedial nucleus of the hypothalamus
DVC: dorsal vagal complex
ER: enrichment ratio
GIP: glucagon-like insulinotropic peptide
GLP-1glucagon-like peptide-1,GLP-1R: glucagon-like peptide-1,GLP-1Rglucagon-like peptide-1 receptor
MBH: mediobasal hypothalamus
NPY: neuropeptide Y
NTS: nucleus of the solitary tract
POMC: pro-opiomelanocortin
PVH: paraventricular nucleus of the hypothalamus
PYY: peptide YY
TH: tyrosine hydroxylase
VMH: ventromedial nucleus of the hypothalamus.

## 1 Introduction

Obesity is a global public health challenge with limited and only mildly efficient non-surgical therapeutic options. Available pharmaceutical interventions produce only a fraction of the weight loss induced by bariatric surgery. One of the most pressing research questions in the field is to understand the mechanisms underlying appetite suppression following bariatric surgery to develop pharmacotherapies mimicking these effects. Several groups have approached this question using combinatorial therapies simultaneously targeting multiple appetite-suppressing pathways. These strategies enable enhanced efficacy through combined activation of independent pathways and/or prolonged suppression of counter-regulatory responses. In fact, the combination of gut-derived peptides (CCK, GLP-1, GIP, and PYY) and/or pancreatic hormones (glucagon and amylin) used at subthreshold doses can produce synergistic reductions in food intake and body weight in rodents [1]. Early clinical forays have also shown benefits for body weight and glucose control with GLP-1/glucagon co-agonists [2,3] and GLP-1/GIP co-agonist [4]. Combinatorial therapies currently represent the most promising strategies to more efficiently treat hyperphagic obesity. However, the mechanisms underlying the synergistic effects of gut/pancreatic hormone combinations are poorly understood. A better characterisation of these mechanisms is needed to design the next generation of drugs targeting the central pathways engaged by combinatorial therapies.

Many successful combinatorial therapies use newly developed GLP-1 receptor (GLP-1R) agonists with enhanced bioavailability and established safety. Chronic administration of GLP-1R agonists reduces food intake and body weight in lean and high-fat diet (HFD)-fed rodents [5]. Due to dose-limiting gastrointestinal side effects, the first GLP-1R agonists elicited modest weight loss in patients with obesity [6,7]; however, later optimised agents (dulaglutide and semaglutide) consistently produced 10% or more body weight loss. Used in combination with glucagon, GIP, or CCK, higher weight loss is achieved at low GLP-1R agonist doses [4,[8], [9], [10], [11]], making GLP-1R agonists widely used in combinatorial therapies currently under investigation.

One gut-derived hormone that has received much less attention in a combinatorial setting is cholecystokinin (CCK), which robustly produces satiation but fails to decrease food intake over 24 h or improve energy balance in chronically dosed rodents due to compensatory increases in meal frequency [12,13]. However, used in combination with GLP-1R agonists, CCKR agonists have therapeutic value in obese rodents. In diet-induced obese rats and genetically obese mice, CCKR and GLP-1R agonists synergistically interact to suppress energy intake and body weight, leading to up to 12% body weight loss [10,14]. In healthy volunteers, a subthreshold dose of CCK increased the hunger score suppressive effect of GLP-1 infusion [15]. However, the underpinning central mechanisms remain unclear. In this study, we investigated the behavioural and neurochemical mechanisms engaged in response to the combined activation of CCK and GLP-1 signalling in mice. To avoid the confounding effects of weight and dietary treatment, we characterised the effects of CCKR and GLP-1R co-agonism in lean mice, and because neuronal activation is best studied in response to an acute stimulus, we assessed this combination's ability to acutely suppress food intake and produce neuronal activation. Using this approach, we found that CCKR receptor agonism can potentiate the anorectic effect of GLP1R agonism at doses that do not produce visceral malaise. We identified the neural substrates engaged in this potentiation and implicated known and novel neurochemical subpopulations in this effect using unbiased molecular profiling of activated neurons. This study demonstrates this approach's ability to decipher the central neurochemical and molecular mechanisms underpinning the anorectic effects engaged in response to combinatorial therapies.

## 2 Materials and methods

### 2.1 Experimental models

All of the studies were conducted in males 9- to 10-week-old rodents at the beginning of the treatments. The rodents were maintained in temperature- and humidity-controlled rooms on a 12 h:12 h light–dark cycle. The animals had free access to water and were fed a standard laboratory chow diet *ad libitum* unless otherwise stated. Food intake studies were conducted on single-housed C57/Bl6 males (Charles River, London, UK) maintained in individually ventilated cages. In all of the studies involving high-fat-fed mice, the mice were maintained on a high-fat diet (HFD) containing 45% energy as fat (#D12266B; Research Diets, Inc.) 3 weeks prior to the beginning of treatments. For pica studies, 8-week-old male Sprague Dawley rats were purchased from Envigo (Frederick, MD, USA). For immunofluorescence studies, we used 8- to 9-week-old *Glp-1r-Cre:R26-tdYFP* males (Richards et al., 2014), *Pomc-GFP* males (stock 009593), and *Npy-GFP* males (stock 006417) purchased from the Jackson Laboratories. All of the scientific procedures on animals were approved and conducted in accordance with the UK Animals (Scientific Procedures) Act 1986.

### 2.2 Compounds

Exenatide and CCK-8 were purchased from Bachem. AC3174 (an exenatide analogue), AC170222 (a CCKR1-selective agonist), and AC170236 (a CCKR2-selective agonist) were synthesised at Amylin Pharmaceuticals and provided via MedImmune/AstraZeneca through its acquisition of Amylin Pharmaceuticals as previously described [10]. AC187 was purchased from Tocris and used at 100 μg/kg.

### 2.3 Compound administration and measurement of the acute feeding response

All of the mice were accustomed to being handled for a minimum of 7 days prior to the beginning of the experiments. Before treatment administration, groups of mice matched for body weight were food deprived for 6 h during the light phase with free access to water. The mice were randomised into treatment groups and received an i.p. injection (10 μL/g of body weight) of either saline, CCK-8 (2.5 or 10 μg/kg), exenatide (1 μg/kg, n = 8), AC3174 (1 μg/kg), AC170222 (2.5 μg/kg), AC170236 (2.5 μg/kg), or a combination of these drugs. Food intake was recorded for the following 2 h. The animals received all of the treatments in a crossover manner. For studies involving pre-treatment with AC187, HFD-fed mice received 10 ml/kg vehicle (saline) or AC187 (100 μg/kg) 30–45 min before receiving either saline or the combination of AC3174 (1 μg/kg) and AC170222 (10 μg/kg).

### 2.4 Meal pattern assessments with BioDAQ cages

The mice were acclimatised for 7 days to BioDAQ cages (BioDAQ E2; Research Diets), randomised into treatment groups, and received 2 i.p. injections (10 μL/g of body weight) per day 1 h after the onset of the light (8 AM) and 1 h before the onset of the dark (6 PM) of either saline, AC3174 (3 μg/kg), AC170222 (30 μg/kg), or combinations of these drugs (3 μg/kg AC3174 + 30 μg/kg AC170222). Body weight was measured daily. Food intake was continuously and automatically recorded for the following 5 days. Eating bouts between 0.01 g and 2 g were included in the data analysis. Meals were defined by an inter-meal interval of at least 300 s and a minimal food consumption of 0.02 g.

### 2.5 Kaolin administration study

Following their acclimatisation week, the male Sprague Dawley rats were given chow and kaolin diets (#K50001; Research Diets). Chow and kaolin were provided in adjacent separate compartments in a divided food hopper. The rats were randomised to appropriate drug treatment groups based on 24 h chow intake, 24 h kaolin intake, and body weight (n = 9). Following an overnight fast in a clean cage, AC3174 and/or AC170222 were i.p. administered at 3 μg/kg and 30 μg/kg, respectively. Cisplatin (#P-4394; Sigma–Aldrich) was i.p. administered at 10 mg/kg as a positive control. Cisplatin was dissolved in sterile saline and sonicated for several minutes. Chow intake and kaolin intake were recorded at 4 h and 24 h post-injection. A final body weight was also recorded at 24 h.

### 2.6 Collection and preparation of brain tissues for immunohistological analysis

The mice were treated as previously described with saline, AC3174 (1 μg/kg), AC170222 (10 μg/kg), or a combination of AC3174 and AC170222. At 80 min after the injection, the mice were anaesthetised and perfuse fixed with PBS and 4% PFA. Their brains were removed and stored in 4% PFA and 30% sucrose at 4 °C for 2 days, then cut into 25 μm coronal sections and preserved at −80 °C in cryoprotectant.

### 2.7 DAB immunohistochemistry

The brain sections were incubated in rabbit anti-c-fos primary antibody (1:8,000; #226003; Synaptic Systems), biotinylated goat anti-rabbit secondary antibody (1:400; #111-065-144; Jackson Laboratories), and ABC solution (#PK-6100; Vector Laboratories) and labelled with DAB solution. Images were obtained using a brightfield slide scanner (Axio Scan Z1; Carl Zeiss Microscopy GmbH) microscope or a light microscope (Olympus BX41; Olympus Corporation) equipped with a digital camera (ColorView; Soft Imaging System). Image analysis and cell counting were manually performed by a blinded experimenter using ImageJ software and the mouse brain atlas from Franklin and Paxinos [16]. For the qualitative c-fos distribution table, the density of c-fos-positive cells was categorised as either absent (−), low (+), moderate (++), high (+++), or very high (++++) following each treatment.

### 2.8 Multiple immunofluorescent labelling

All of the brain sections were incubated with c-fos primary antibody (1:2,000; #226003; Synaptic Systems), biotinylated goat anti-rabbit secondary antibody (1:800; #111-065-144; Jackson Laboratories), and streptavidin (1:500; AlexaFluor 405 #S32351; AlexaFluor 594 #S11227; Life Technologies). The brain sections were then incubated in either mouse anti-TH primary antibody (1:100; #22941; Immunostar) and goat anti-mouse secondary antibody (1:400; DyLight 405 #115-475-146; Jackson Laboratories), chicken anti-GFP primary antibody (1:1000; #AB13970; Abcam), goat anti-chicken secondary antibody (1:500; AlexaFluor 488 #AB150173; Abcam), chicken anti-RFP (1:1,000; #600-901-379; Rockland), goat anti-chicken secondary antibody (1:500; DyLight 594 #SA5-10072; Thermo Fisher Scientific), or rabbit antibody anti-pS6 240/244 Alexa Fluor 594 conjugate (1:200,#9468; Cell Signalling Technology).

### 2.9 Tissue imaging and analysis

Images were obtained using a confocal microscope (Leica TCS SP8; Leica Microsystems CMS GmbH). Image analysis and cell counting were performed semi-automatically using HALO software (HALO; Indica Labs) and the mouse brain atlas from Franklin and Paxinos [16] was used for neuroanatomical reference.

### 2.10 PhosphoTRAP

We used the PhosphoTRAP assay as previously described to identify transcripts enriched in neurons activated or inhibited by the co-administration of AC3174 and AC710222 [17].

For the sample preparation, the mice were treated as previously described with saline or the combination of AC3174 and AC170222. At 30 min post-injection, the mice were sacrificed by cervical dislocation and their brain micro-dissected in buffer B (2.5 mM of HEPES at pH 7.4, 4 mM of NaHCO_3_, 35 mM of glucose, and 100 mg/mL of cycloheximide in methanol) on ice under 10× magnification to collect the MBH and dorsal vagal complex (DVC: area covering the area postrema, nucleus of the solitary tract). MBH and DVC tissues of 4 mice were pooled into 1 MBH sample and 1 DVC sample, leading to 4 samples by experimental conditions that were homogenised in buffer C (10 mM of HEPES at pH 7.4, 150 mM of KCl, 5 mM of MgCl_2_, 100 nM of calyculin A, 2 mM of DTT, 100 U/mL of RNasin, 100 mg/mL of cycloheximide, and Roche protease and phosphatase inhibitor cocktails) and clarified by centrifugation at 2,000 g at 4 °C for 10 min. Supernatants were resuspended in 1 ml of 10% NP40 and 10 μL of 1,2-diheptanoyl-sn-glycero-3-phosphocholine (DHPC), incubated for 2 min on ice, and centrifuged at 16,100 g at 4 °C for 10 min. Then 25 μL of the sample was used for RNA extraction (input sample). The remaining sample was used for ribosome immunoprecipitation as described as follows.

For ribosome immunoprecipitation, Protein A Dynabeads (Invitrogen) were washed 3 times with buffer A (10 mM of HEPES, 150 mM of KCl, 5 mM of MgCl_2_, and 1% NP40 at pH 7.4) before incubation with anti-pS6 240/244 antibody (#2215, Cell Signalling Technology) (4 μg per ip sample) and 0.1% bovine serum albumin (BSA) in buffer A (300 μL per i.p. sample). Antibody-bead conjugates were mixed end over end at 4 °C overnight. Then 300 μL of antibody-bead conjugates per i.p. were washed twice with wash buffer D (10 mM of HEPES, 350 mM of KCl, 5 mM of MgCl_2_, 2 mM of DTT, 1% NP40, 100 U/mL of RNasin, 100 mg/mL of cycloheximide, and Roche protease and phosphatase inhibitor cocktails). Antibody-bead conjugates were resuspended in 200 μL of homogenisation buffer C supplemented with 50 μL of 10% NP40 and 10 μL of DHPC for 1 mL of buffer C and 1 μM of ZK10 (a gift from Dr. Zackary Knight). The remaining supernatant was added to the antibody-beads conjugates, resuspended by pipetting and placed on end over end at 4 °C for 10 min. The beads were washed 4 times with 0.9 mL of ice cold wash buffer D and resuspended in 350 μL of RLT buffer (i.p. sample).

For RNA extraction, RNA was extracted using an RNeasy Micro kit (#74004; Qiagen). RNA quantity was assessed using a Quant-iT RiboGreen RNA Assay kit (#R11490; Thermo Fisher Scientific), and the samples’ RNA quality was assessed using Pico chips on an Agilent Bioanalyser. The samples were cleared from DNA contamination using a TURBO DNA-free kit (#AM1907; Thermo Fisher Scientific).

For cDNA preparation and sequencing, cDNA was prepared using a Smart Seq v4 Ultra Low Input RNA kit (#634889; Clontech). Library preparation was done using a Nextera XT kit (Illumina), and the samples were run on a NexSeq 500 System (#FC-131-1024; Illumina).

For differential enrichment ratio calculations, for each sample, we first calculated the enrichment ratio (ER) of the i.p. sample over the input sample (FPKM_ip_/FPKM_input_). We then calculated the differential enrichment ratio (dER) for each transcript by dividing the mean ER in the treatment (AC3174 + AC170222) group by the mean ER in the control (saline-treated) group. The dER was used to identify transcripts enriched in neurons activated or inhibited by the AC3174 + AC170222 treatment. Sequencing data are available on GEO (number pending).

### 2.11 Tissue preparation for multiplexed in situ hybridisation

Brains were removed at termination and snap-frozen on crushed dry ice and stored at −80 °C until cryosectioning. The brains were divided into forebrains and hindbrains at the level of the pons and mounted on a pre-cooled cryostat sample holder with Tissue-Tek O.C.T. Compound (Sakura Finetek). Series of 12 μm thick coronal sections were cut on a cryostat (CM3050 S; Leica) and collected on Superfrost Plus microscope slides (Thermo Fisher Scientific) and stored at −80 °C until use. Serial sections were cut through the ARH with a sampling distance of 216 μm, collecting approximately 5 samples per animal. Similarly, serial sections were cut through the AP and NTS with a sampling distance of 72 μm, collecting approximately 5–8 samples per animal from each region.

### 2.12 RNAscope fluorescent multiplex in situ hybridisation

RNAscope in situ hybridisation was performed on brain cryosections using an RNAscope Fluorescent Multiplex Assay V2 (Advanced Cell Diagnostics) to simultaneously visualise up to 3 mRNA targets using mouse-specific probes directed against genes listed in Supplementary Table 8. A 3-plex positive control probe targeting the housekeeping genes Polr2a, PPIB, and UBC (cat. no. 320881; Advanced Cell diagnostics) was used to verify the mRNA quality and assay procedures. A 3-plex probe targeting the bacterial gene dapB (cat. no. 320871), which is not expressed in eukaryotes, was used as a negative control. Slides with cryosections were treated according to the RNAscope Fluorescent Multiplex Assay user manual using FITC, Cy3, and Cy5 as fluorophores. After in situ hybridisation, the slides were counterstained with DAPI, cover-slipped, and scanned under a 20× objective in an VS-120 fluorescent slide scanner (Olympus).

### 2.13 Statistical analysis

All of the treatments were systematically randomly assigned and administered to weight-matched groups of mice by a blinded researcher. For feeding, body weight responses, and histological analyses, data were analysed using a two-way ANOVA corrected for multiple comparisons and an alpha risk of 0.05. To identify the transcripts enriched in activated or inhibited neurons in response to each treatment, we first filtered the dataset and kept the transcripts with an average expression in the input samples greater than or equal to 2 FPKM. Statistical analyses were performed on ER values (see Methods) and treatment groups were compared using one-tailed unpaired Student's t-test with an alpha risk of 0.01.

## 3 Results

### 3.1 Subthreshold GLP-1R agonism acutely potentiated the anorectic response to CCK in lean and HFD-fed mice

**Figure 1:**
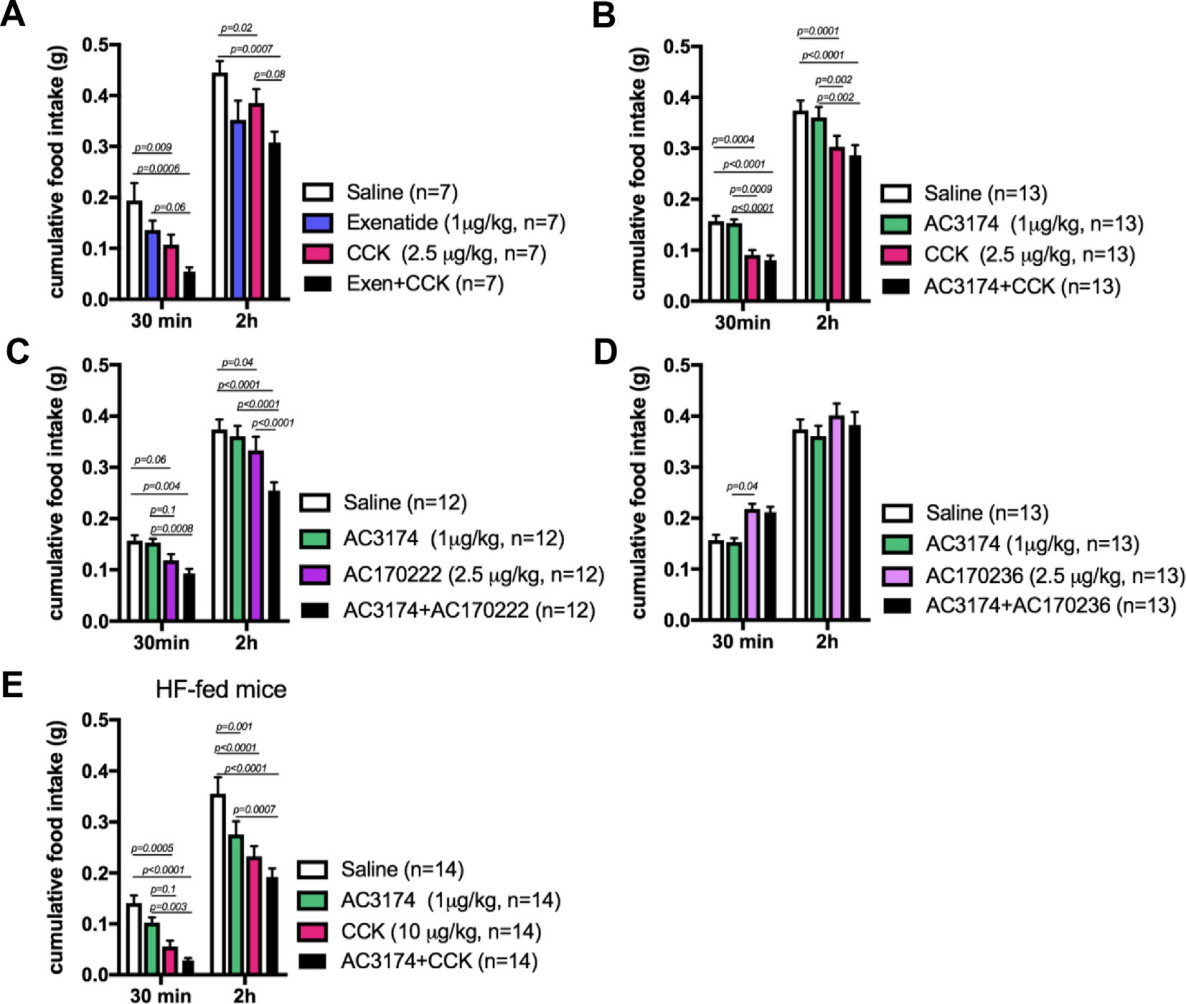
A subthreshold dose of GLP-1R agonist AC3174 acutely increased the anorectic response to CCKR1 agonism. Two h food intake in response to an i.p. injection of saline, CCK-8, exenatide, GLP-1R agonist AC3174, CCK1R agonist AC170222, CCK2R agonist AC170236, or an equimolar combination of these drugs in the lean chow-fed mice (A–D) or the mice fed a HF diet for 3 weeks (E). Data (means ± sem) were analysed using 2-way ANOVA for repeated measures with 2 factors (treatment and time) and Tukey's multiple comparison tests. The mice were 9 weeks old at the beginning of the studies (±26 g).

We first tested the effect of a subthreshold dose of exenatide [18], a well-characterised GLP-1R agonist, on the acute anorectic effect of CCK-8 in lean mice. As expected, CCK-8 rapidly reduced dark-onset food intake. The subthreshold dose of exenatide slightly reduced food intake alone and potentiated CCK-8-induced anorexia at 30 min (Figure 1A). We then tested whether AC3174, an exenatide analogue, would replicate this effect. AC3174 is a C-terminal amidated peptide that is 39 residues in length and is an analogue of natural peptide exendin-4 with leucine substituted for methionine at position 14, Leu (14) exendin-4. It displays similar in vitro and in vivo efficacy compared to exendin-4 in rodent models [19,20]. We found that a subthreshold dose of AC3174 (Supplementary Fig. 1a) did not enhance the anorectic effect of CCK-8 (Figure 1B).

CCK1R signalling has been shown to mediate the anorectic effect of CCK [21]. We therefore tested whether CCK1R signalling was mediating the effect of the CCK + AC3174 combination. We combined AC3174 with novel isoform-specific agonists for the CCK1R or CCK2 receptors, AC170222 and AC170236, respectively [19]. AC170222 is 4,000-fold more selective for CCK-1R than CCK-2R, and AC170236 is 6,000-fold more selective for CCK-2R than CCK-1R [10]. AC3174 potentiated the acute anorectic effect of AC170222 (Figure 1C). In contrast, AC170236 failed to suppress dark-onset food intake and in combination with AC3174 did not produce anorexia (Figure 1D). Importantly, neither AC3174, AC170222, or their combination produced pica behaviour (Supplementary Fig. 2). Thus, a subthreshold dose of a GLP-1R agonist (exenatide or AC3174) potentiated the acute anorectic effect of a CCK1R agonist (CCK-8 or AC170222) in lean mice.

We then tested whether the response to CCK1R and GLP-1R co-agonism was maintained in mice fed a high-fat (HF) diet (Supplementary Fig. 1b), a condition associated with reduced sensitivity to the satiating effects of CCK and GLP-1 (Williams et al*.*, 2011; Frank A. Duca, Sakar, and Covasa, 2013). We confirmed that the HF diet blunted the anorectic response to CCK (Supplementary Fig. 1c and d) as the dose required to produce satiation was 5 times higher than in the chow-fed mice. We observed a trend toward a potentiated anorectic response to the coadministration of a subthreshold dose of AC3174 (Supplementary Fig. 1e) with CCK-8 in the HF-fed mice (Figure 1E; *p* = 0.08 at 30 min and *p* = 0.12 at 2 h).

### 3.2 Chronic GLP-1R and CCK1R co-agonism produced sustained hypophagia

**Figure 2:**
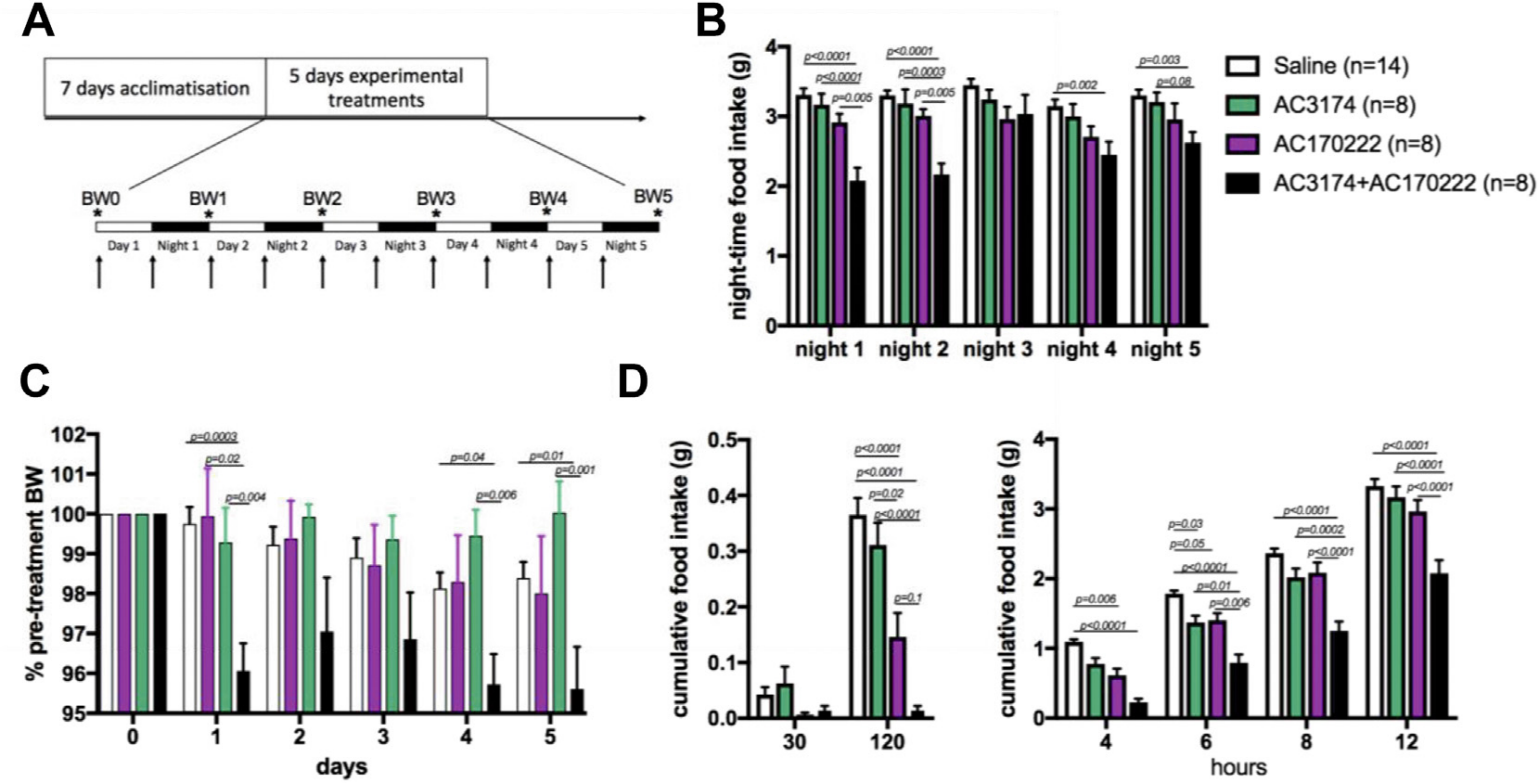
Chronic GLP-1R and CCK1R co-agonism produced sustained hypophagia and weight loss. The mice were treated with i.p. administration of saline, GLP-1R agonist AC3174 (3 μg/kg), CCK1R agonist AC170222 (30 μg/kg), or AC3174 + AC170222 twice daily for 5 days. Arrows indicate injections and stars indicate times at which body weight was measured (A). Night-time food intake (B), weight loss (C), and hourly night-time food intake during night 1 (D). Data (means ± sem) were analysed using 2-way ANOVA for repeated measures with 2 factors (treatment and time) and Tukey's multiple comparison tests. The mice were 9 weeks old at the beginning of the studies (±26 g).

Previous studies indicated that chronic co-administration of GLP-1R and CCK1-R agonists produced sustained decreases in food intake and body weight in obese rats [10]. We tested whether this was also the case in lean mice by administering the compounds twice daily for 5 days using higher doses than in the acute study, which still to produce pica behaviour (Supplementary Fig. 2 and Figure 2A). Both mono-agonists AC3174 and AC170222 failed to decrease food intake and weight gain over the 5 days of treatment when given alone. In contrast, the drug combination produced a decrease in night-time food intake and body weight that was sustained over the 5 days of the study (Figure 2B,C). To characterise the behavioural mechanisms engaged by the treatments to suppress food intake, we used BioDAQ cages to monitor meal size and meal frequency over 5 days in the mice exposed to the drugs twice daily.

The GLP-1R agonist AC3174 moderately reduced food intake during the first 6 h of the dark phase on the first day of treatment but not at the later time points (Figure 2D). This transient reduction in food intake was mediated by a trend for decreased meal size (Supplementary Fig. 3a), and the meal number remained unaffected (Supplementary Fig. 3b). The CCK1R agonist AC170222 produced a rapid and substantial reduction in food intake during the first 6 h following the first injection but this effect was also transient (Figure 2D). This acute reduction in food intake was mediated by a transient reduction in meal size and meal frequency between 2 h and 6 h after the first injection (Supplementary Fig. 3). Administered in combination, AC3174 and AC170222 produced a greater suppression of food intake starting 2 h after the injections and this interaction continued during for 12 h following the first injection (Figure 2D). Reduced food intake was associated with a rapid and transient decrease in meal size and a sustained decrease in meal frequency (Supplementary Fig. 3). Thus, not only were the compounds interacting to produce a greater anorectic effect, the combination also produced sustained anorexia at time points when the single compounds ceased to be effective.

### 3.3 Subthreshold GLP-1R agonism potentiated the neuronal activation induced by CCK1R agonism only in a few discrete brain sites

**Figure 3:**
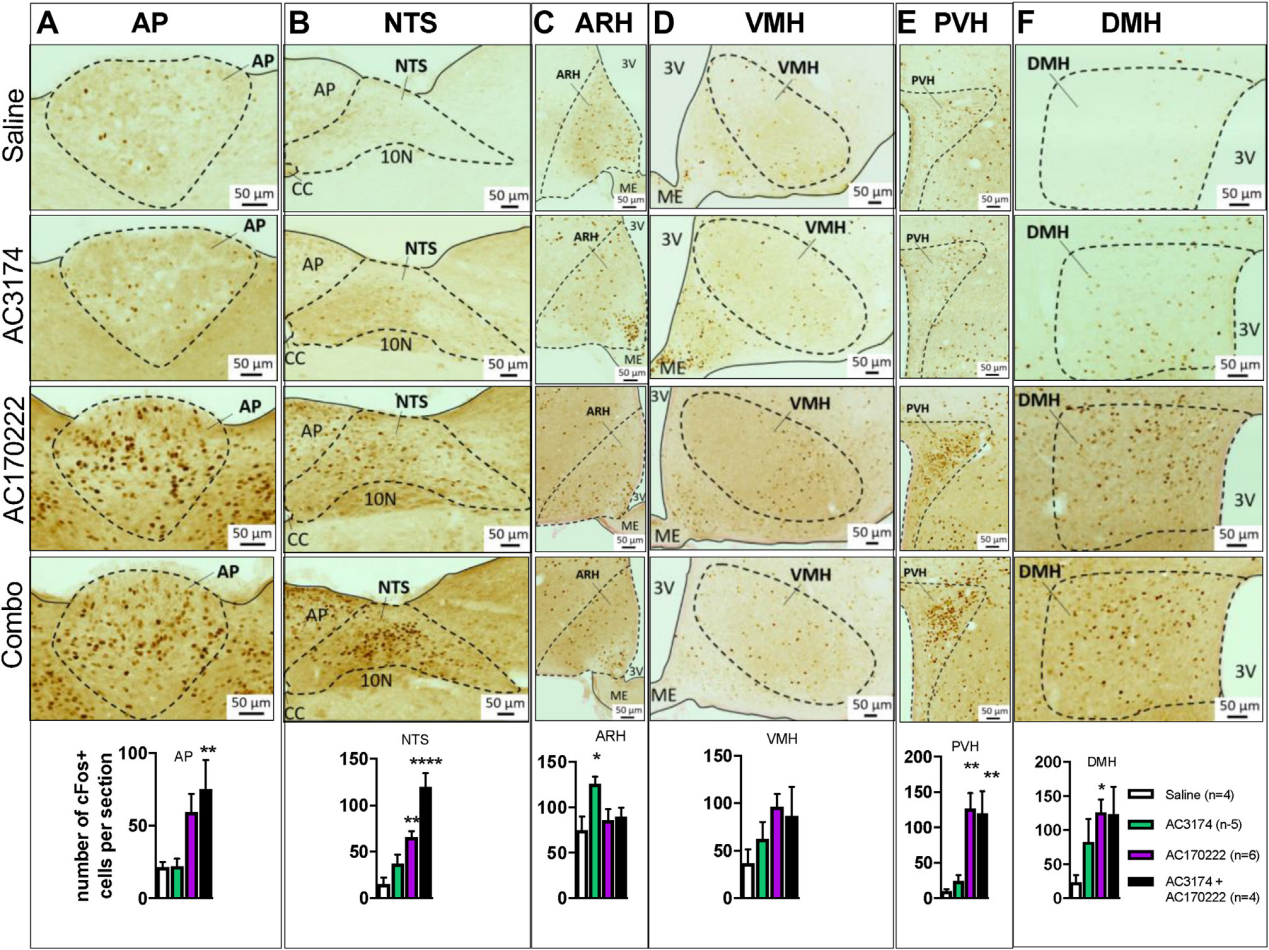
Neuronal activation in response to acute GLP-1R and CCK1R co-agonism in hypothalamic and caudomedial hindbrain sites. C-fos immunolabelling in the AP (A), NTS (B), ARH (C), VMH (D), PVH (E), and DMH (F) 80 min after an i.p. administration of saline, GLP-1R agonist AC3174, CCK1R agonist AC170222, or AC3174 + AC170222. Data are means ± sem. ∗*p* < 0.05, ∗∗*p* < 0.01, and ∗∗∗∗*p* < 0.0001 vs saline. The mice were 9 weeks old at the beginning of the studies (±26 g). A minimum of 4 sections per mouse per brain region were analysed.

We used the acute administration protocol (Figure 1) to characterise the neural responses to GLP-1R + CCK1R co-agonism in the lean mice. We first qualitatively assessed neuronal activation throughout the neuraxis using c-fos immunostaining (Supplementary Table 1). The GLP1-R agonist AC3174 alone induced a moderate increase in neuronal activation in a few brain areas. The CCK1R agonist AC170222 produced more robust neuronal activation and activated a higher number of brain sites, both distinct and overlapping with the sites activated by AC3174. In response to the drug combination, neuronal activation was higher than with the monotherapies in a few brain sites including the central amygdala (CeA), dorsolateral parabrachial nucleus (DLPBN), and nucleus of the solitary tract (NTS). However, the most robust activation was observed in the NTS (Supplementary Fig. 4 and Figure 3). Intriguingly, although AC3174 produced robust neuronal activation in the arcuate nucleus of the hypothalamus (ARH), the drug combination failed to activate the ARH compared to the control group.

We then quantified the number of c-fos-positive cells in several hindbrain and hypothalamic sites including the ARH, ventromedial, dorsomedial, and paraventricular nuclei of the hypothalamus (VMH, DMH, and PVH), area postrema (AP), and NTS. This quantitative assessment confirmed the qualitative results (Supplementary Table 1). The combination treatment did not produce more neuronal activation than the monotherapies in the hypothalamic sites or AP but did produce more neuronal activation in the NTS (Figure 3A–F). Again, in the ARH, the combination treatment produced less neuronal activation than AC3174 alone (Fig. 3C).

### 3.4 Neurochemical characterisation of the neurons responding to the combination of GLP-1R/CCK1-R agonists

The dorsal vagal complex (DVC) is a primary sensory relay for gut-derived satiation signals. In particular, DVC POMC and TH neurons have been implicated in the response to peripheral CCK [22], whereas the anorectic response to GLP-1R agonists relies on the activation of ARH POMC neurons [23]. Based on these data and the fact that the DVC and ARH are two critical brain sites mediating the control of food intake, we focused all of the subsequent studies on these two brain sites to identify the neuronal populations engaged by GLP-1R + CCK1R pharmacological treatment. We first performed a candidate-driven assessment of the neurochemical populations responding to the compounds.

**Figure 4:**
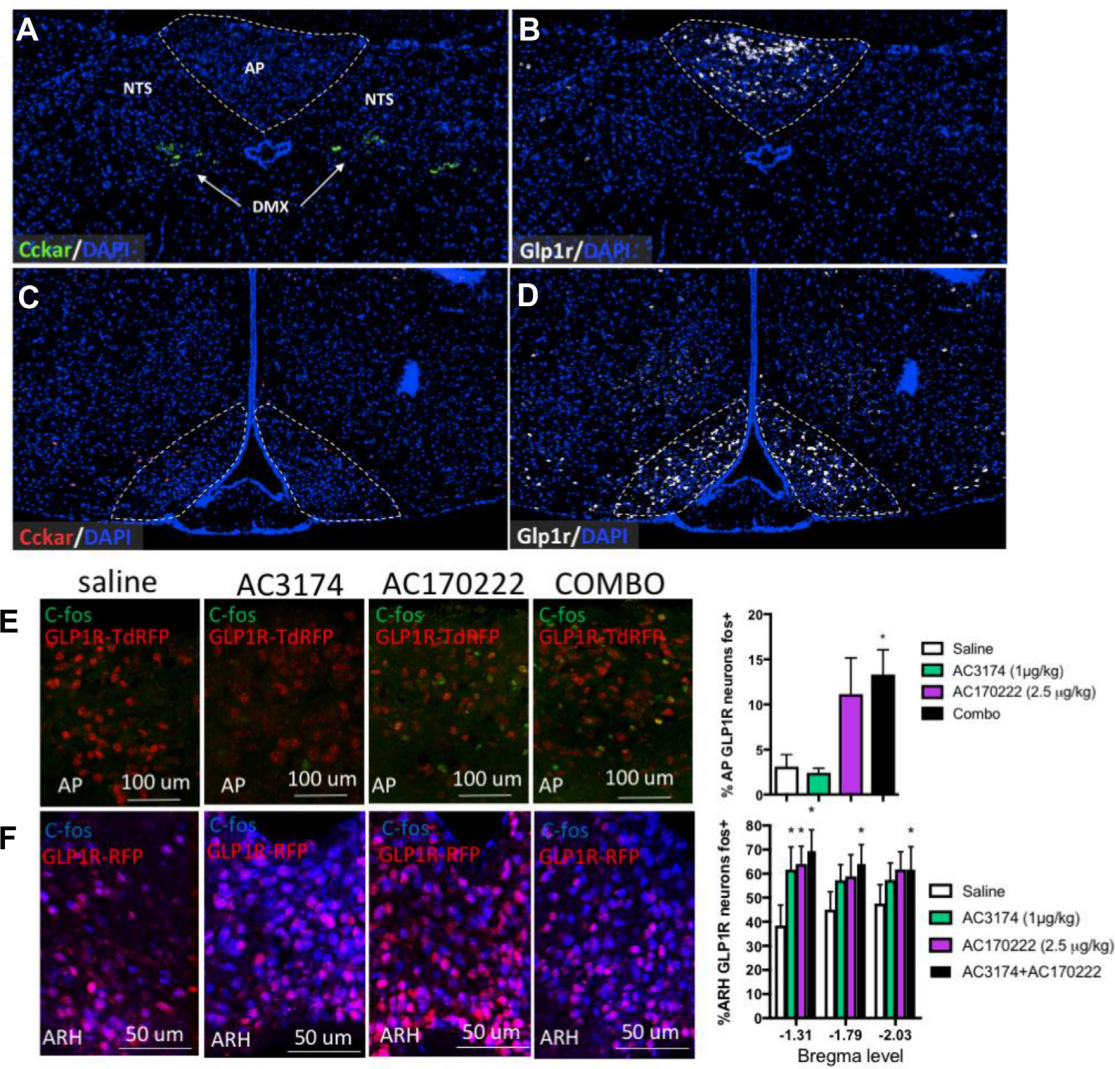
GLP-1R and CCK1R co-agonism did not integrate within CCK1R or GLP-1R neurons. Representative images showing *Cckar* (A and D) and *Glp-1r* (B and C) distribution in the AP, NTS, and ARH. Representative images and quantification of neuronal activation in AP (E), ARH (F), or GLP-1R neurons in response to an i.p. administration of saline, GLP-1R agonist AC3174 (1 μg/kg), CCK1R agonist AC170222 (2.5 μg/kg), or AC3174 + AC170222 (1 μg/kg + 2.5 μg/kg). Data are means ± sem. ∗*p* < 0.05 vs saline.

Given that both GLP-1R and CCK1R are expressed in the DVC and ARH, we first considered the possibility that the integration between GLP-1R and CCK1R signalling could occur at a single cell level in cells expressing both of the receptors that could directly mediate the feeding-suppressive effect. We used RNAscope to determine the degree of co-expression of *Glp1r* and *Cckar* in the DVC and ARH. In the DVC, there were virtually no cells co-expressing *Cckar* and *Glp1r* as most *Cckar* signals localised to the DMX (Figure 4A), whereas most *Glp1r* signals localised to the AP (Figure 4B) as previously reported [24]. In the MBH, *Glp1r* signals were abundant and concentrated in the ARH (Figure 4C), whereas *Cckar* signals were rare and scattered (Figure 4D). Collectively, these results did not support the possibility that GLP-1R and CCK1R signalling converge at a single cell level. Instead, they suggest that the interaction between the two compounds occurred at a neuronal network level.

Another possible mechanism mediating the integration of GLP-1R and CCK1R signalling would be that circuits engaged downstream of CCK1R signalling modulate neuronal activation of GLP-1R neurons, increasing activation of GLP-1R neurons in the DVC and decreasing neuronal activation of GLP-1R neurons in the ARH. To test this, we used reporter mice expressing tdRFP under the control of GLP-1R [24] and quantified neuronal activation in GLP-1R-expressing neurons in response to acute peptide administration as shown in Figure 1. Surprisingly, the GLP-1R agonist AC3174 failed to activate GLP-1R neurons in the AP at the dose we used (Figure 4E). In contrast, the CCK1R agonist AC170222 activated ∼10% of AP GLP-1R neurons (Figure 4E), but the co-administration of AC3174 and AC170222 did not engage a higher number of GLP-1R neurons in the DVC. In the ARH, AC3174 and AC170222 alone or in combination robustly activated GLP-1R neurons with no substantial difference between the treatments (Figure 4F). Thus, co-activation of GLP-1R and CCK1R signalling did not integrate in the DVC or ARH GLP-1R neurons.

**Figure 5:**
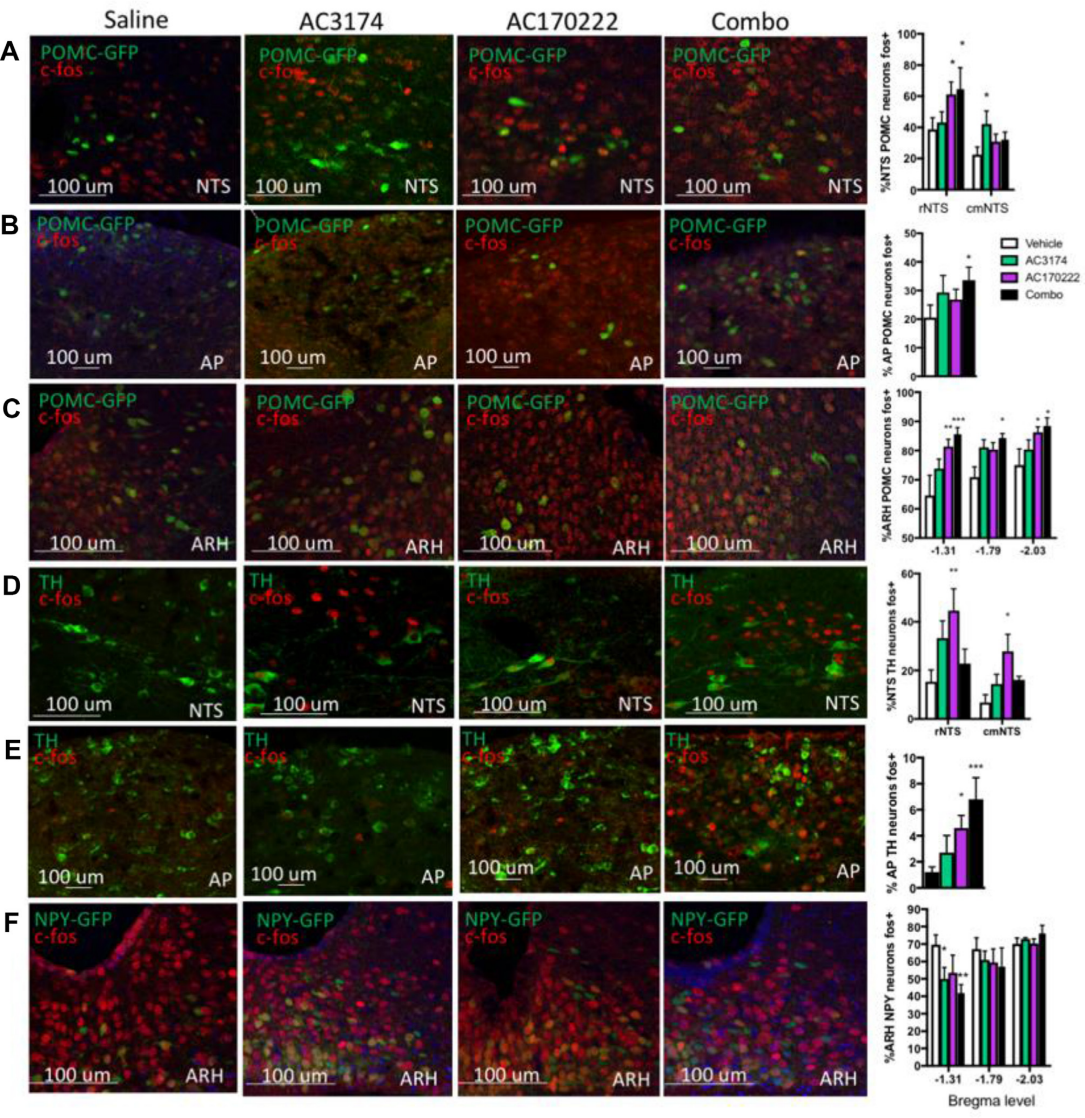
Neurochemical characterisation of the neuronal subpopulations activated by the combination of GLP-1R and CCK1R agonists. Representative images and quantification of neuronal activation in NTS POMC (A), AP POMC (B), ARH POMC (C), NTS TH (D), AP TH (E), and ARH NPY (F) neurons in response to an i.p. administration of saline, GLP-1R agonist AC3174 (1 μg/kg), CCK1R agonist AC170222 (2.5 μg/kg), or AC3174 + AC170222 (1 μg/kg + 2.5 μg/kg). Data are means ± sem. ∗*p* < 0.05, ∗∗*p* < 0.01, and ∗∗∗*p* < 0.001 vs saline. rNTS: rostral NTS; cmNTS: caudomedial NTS.

A last possible scenario would involve the contribution of a distinct cell population located downstream from both CCK1R and GLP-1R signalling and integrating inputs from GLP-1R and CCK1R neurons. Candidate neuronal populations that could be involved include DVC POMC and TH neurons [25] and ARH POMC neurons [23]. We examined neuronal activation in these neuronal populations using POMC-GFP mice and immunolabelling against TH. NTS POMC neurons were activated both by AC3174 and AC170222; however, the combination treatment did not further activate this neuronal population (Figure 5A). AP POMC neurons were not significantly activated by the drugs administered alone, whereas the combination produced a slight but statistically significant increase in the activation of AP POMC neurons (Figure 5B). In the ARH, co-treatment induced significantly more activation of POMC neurons than monotherapies (Figure 5C). NTS TH neurons were activated by both AC3174 and AC170222 alone but the combination treatment did not further activate this neuronal population (Figure 5D). In the AP, co-treatment with GLP-1R and CCK1R agonists produced significantly more activation of TH neurons (Figure 5E). We then assessed the treatment's effect on the activation status of ARH AGRP neurons in NPY-GFP mice. Combinatorial therapy produced significantly more inhibition of ARH AGRP neurons in the rostral ARH than the monotherapies. Collectively, these data indicated that GLP-1R and CCK1R co-agonism converged and were integrated in AP POMC and TH neurons and ARH POMC and AGRP neurons. However, these experiments failed to identify the neuronal population involved in the increased neuronal activation produced by the GLP-1R/CCK1R combinatorial therapy in the NTS. We therefore used an unbiased approach for the molecular profiling of neurons specifically activated by the AC3174/AC170222 combination.

### 3.5 Unbiased molecular characterisation of neurons activated by combined GLP-1R/CCK1R agonism using PhosphoTRAP

We used PhosphoTRAP [17] to generate expression profiles from activated and inhibited neurons in the mediobasal hypothalamus (MBH) and DVC in response to AC3174 and AC17022 administered alone or in combination. This method takes advantage of the fact that ribosomal protein S6 is phosphorylated in activated neurons in response to an acute stimulus. Using immunoprecipitation (ip), phosphorylated ribosomes can be captured from mouse brain homogenates, thereby enriching mRNA selectively expressed in neurons activated by the stimulus. Using differential transcriptomic of immunoprecipitated phosphorylated ribosomes against total RNA, molecular markers for activated and inhibited cells can be identified.

We confirmed that in response to our treatments, c-fos expression correlated with the phosphorylation of ribosomal protein S6 (Supplementary Fig. 5), allowing us to use immunoprecipitation against pS6 to enrich for mRNA expressed in activated neurons. We exposed mice to the 4 treatments, micro-dissected the MBH and DVC, and extracted and sequenced RNA from whole homogenates or immunoprecipitated pS6 ribosomes. For each transcript, we calculated the enrichment ratio in the ip sample (ER) and corrected it for the average ER measured in control samples (dER) (see Methods). We validated the neuroanatomical specificity of MBH and DVC dissections and confirmed that mRNA from immunoprecipitated samples was enriched in markers of neuronal activation (Supplementary Fig. 6).

We obtained an unbiased molecular signature of MBH and DVC neurons activated or inhibited by AC3174, AC170222, and AC3174 + AC170222 (Supplementary Tables 2-7). In the DVC and MBH, the molecular profiles of neurons activated or inhibited between conditions were quite distinct, with only 20–30% of overlapping genes between conditions, reinforcing the idea that AC3174, AC170222, and their combination engage mostly distinct neuronal populations (Supplementary Fig. 7 and 8). We focused our analysis on genes selectively identified as markers of neurons responding to the combinatorial treatment but not the monotherapies and genes identified as enriched in neurons modulated by the combinatorial treatment vs monotherapy (defined as follows: *p* value for combo vs AC3174 < 0.05 and *p* value for combo vs AC170222 < 0.05).

**Figure 6:**
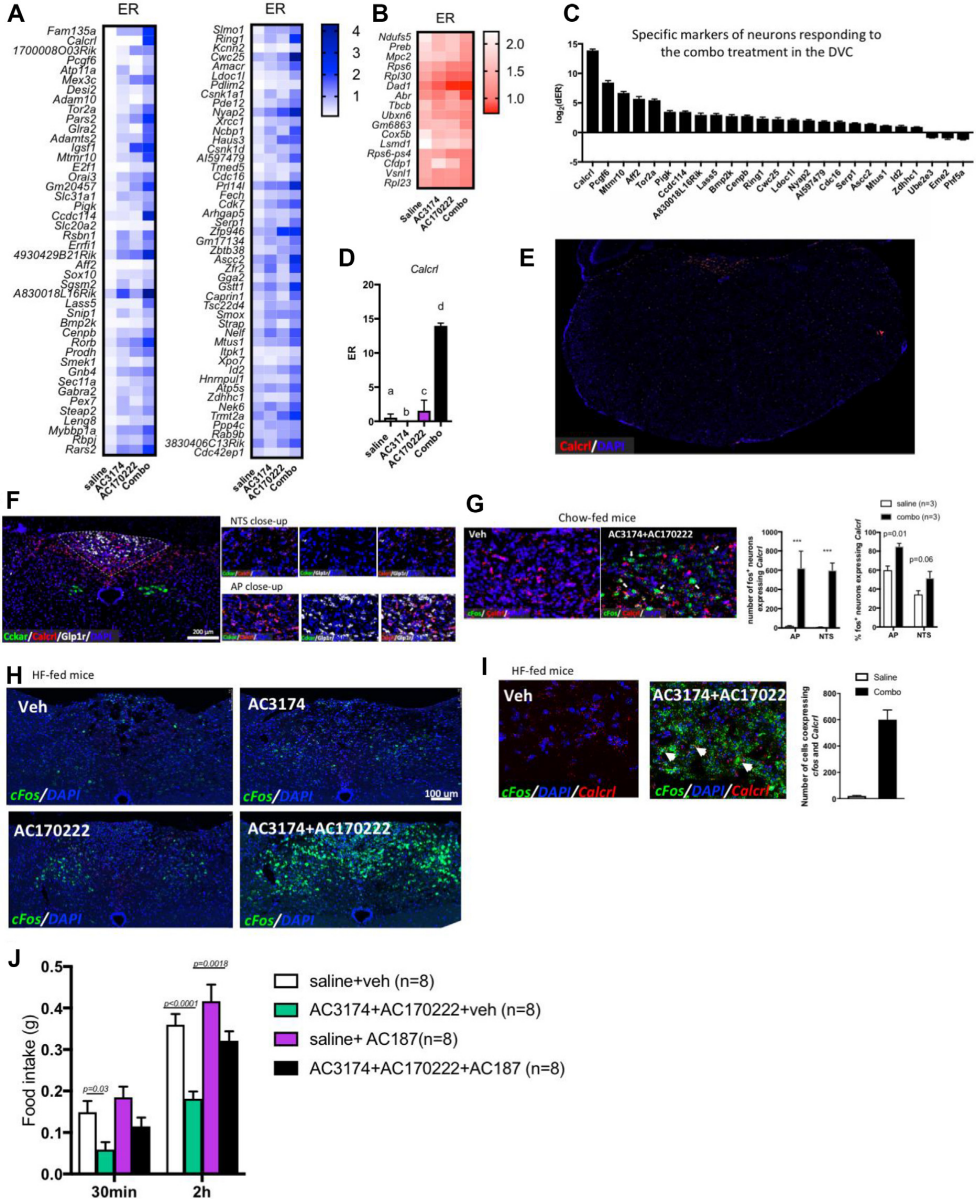
PhosphoTRAP assay identified the molecular profile and neurons activated or inhibited by the combinatorial treatment in the DVC. Enrichment ratios of transcript significantly (*p* < 0.01) enriched in neurons activated (A) and inhibited (B) by co-treatment with GLP-1R agonist AC3174 and CCK1R agonist AC170222, but not in neurons activated or inhibited by the monotherapies (AC3174 vs saline: *p* > 0.1 and AC170222 vs saline: *p* > 0.1). Differential enrichment ratios (C) of transcripts enriched in neurons more activated by the combinatorial therapy than monotherapies (AC3174 vs combo: *p* < 0.05 and AC170222 vs combo: *p* < 0.05). Differential enrichment ratios of *Calcrl* (D). Expression of *Calcrl* in the caudal medulla (E). Co-localisation of *Cckar* (green), *Glp1r* (white), and *Calcrl* (red) in the DVC (F). Co-localisation of c-fos (green) and *Calcrl* (red) in response to i.p. administration of the vehicle (left) or combinatorial treatment in the chow-fed mice (right) (G). Expression of c-fos (green) (H) and co-localisation of c-fos (green) and *Calcrl* (red) (I) in response to i.p. administration of the vehicle, AC3174, AC170222, or combinatorial treatment in the high-fat-fed mice. Feeding response to saline or AC3174 (1 μg/kg) + AC170222 (10 μg/kg) in the mice pre-treated with vehicle or AC187 (100 μg/kg) (J). Data are means ± sem. Means sharing a letter are not statistically different.

In the DVC, 254 genes were significantly enriched or depleted in activated ribosomes of the mice treated with AC3174 + AC170222 (*p* < 0.01) compared to controls (Supplementary Table 4). Of these, 45 genes were enriched (Figure 6A) and 26 were depleted (Figure 6B) selectively in response to the combination treatment but not the monotherapies (AC3174 vs combo and AC170222 vs combo: *p* > 0.1).A total of 25 genes were more enriched or depleted in response to the combination treatment vs the monotherapies (Figure 6C and Supplementary Fig. 7). To identify druggable pathways, we focused our analysis on genes expressing plasma membrane or secreted protein. Calcitonin receptor-like receptor (*Calcrl*) was enriched 15-fold in neurons activated in response to AC3174 + AC170222 and not enriched in neurons responding to the monotherapies and therefore was further investigated as a marker for neurons responding specifically to the combination treatment. *Calcrl* encodes the core receptor component for CGRP, a neurotransmitter implicated in the physiological control of food intake in rodents [26,27]. Using RNAscope, we determined the distribution of *Calcrl*^+^ neurons in the DVC, the expression of *CCKar* and *Glp1r* in *Calcrl*^*+*^ neurons, and the expression of c-fos in DVC *Calcrl*^+^ neurons in response to GLP-1R and CCK1R co-agonism. We found that *Calcrl* was concentrated in the AP and AP/NTS border and absent from the rest of the medulla (Figure 6E). While the majority of AP *Calcrl*^*+*^ neurons expressed *Glp1r*, NTS *Calcrl* neurons expressed neither *Glp1r* or *Cckar* (Figure 6F). In response to GLP-1R and CCK1R co-agonism, 85% of AP c-fos^+^ neurons and 50% of NTS c-fos^+^ neurons expressed *Calcrl* (Figure 6G). Then we examined hindbrain sections from mice maintained on a HF diet and treated with vehicle, AC3174, and AC170222 alone or in combination (Figure 1E) and processed for RNAscope against c-fos and *Calcrl*. GLP-1R and CCK1R co-agonism significantly increased neuronal activation in the AP and NTS compared to both mono-agonists, and a majority of the neurons activated by the combinatorial treatment expressed *Calcrl*^.^(Figure 6H,I).These results confirmed that *Calcrl* is a marker for neurons activated by the GLP1R/CCKR combinatorial treatment in the chow- and HF-fed mice.

To confirm the functional relevance of these findings, we tested the effect of calcitonin-like receptor (CLR) blockade on the anorectic response to GLP-1R and CCK1R co-agonism in the mice fed a HF diet. We used the amylin receptor antagonist AC187, which also blocks calcitonin receptor (CT) and CLR signalling, at a dose that did not affect feeding alone. This compound does not distinguish well between amylin and CT receptors and is less effective at blocking CLR signalling. Pre-treatment with AC187 blunted the acute anorectic response to AC3174 + AC170222, indicating that targets of AC187 including CLR mediated the feeding-suppressive effect of GLP-1R and CCK1R combination therapy (Figure 6J). Of note, although AC187 antagonises CT, the vast majority of NTS CT neurons are concentrated in the most ventral portion of the caudomedial NTS [28], whereas neurons responding to combination therapy are absent from this area and mostly occupy the dorsal NTS and AP/NTS border (Figure 6H). In addition, a large proportion of NTS CT neurons co-express TH, and these results showed that NTS TH neurons are not further activated by GLP-1R and CCK1R co-agonism (Figure 5D). Thus, it was unlikely that CT antagonism by AC187 accounted for the blockade of the anorectic response to GLP-1R and CCK1R co-agonism in response to AC187 pre-treatment.

**Figure 7:**
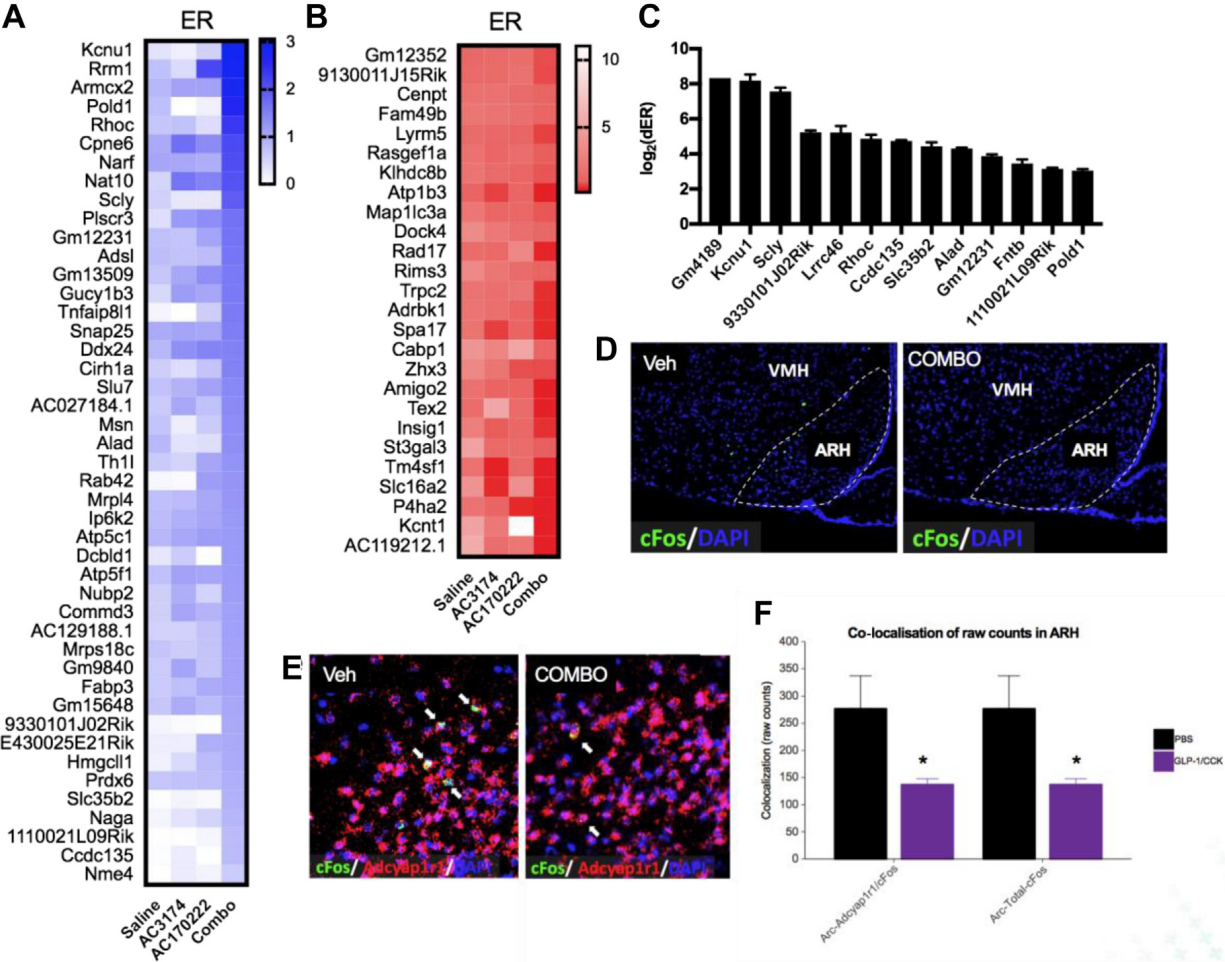
PhosphoTRAP assay identified the molecular profile and neurons activated or inhibited by the combinatorial treatment in the MBH. Enrichment ratios of transcripts significantly (*p* < 0.01) enriched in neurons activated (A) and inhibited (B) by co-treatment with the GLP-1R agonist AC3174 and CCK1R agonist AC170222, but not in neurons activated or inhibited by the monotherapies (AC3174 vs saline: *p* > 0.1 and AC170222 vs saline: *p* > 0.1). Differential enrichment ratios (C) of transcripts with higher enrichment in response to the combinatorial therapy than the monotherapies (AC3174 vs combo: *p* < 0.05 and AC170222 vs combo: *p* < 0.05). RNAscope ISH against c-fos (D and F) and c-fos + *Adcyap1r1* (E and F) in the ARH of the mice treated with the combinatorial treatment vehicle. Data are means ± sem. ∗*p* < 0.05.

We also used the PhosphoTRAP assay to obtain the molecular signature of neurons in the MBH regulated by the combinatorial treatment (Figure 7 and Supplementary Fig. 8). Given that the overall response we observed in the MBH indicated that GLP-1R and CCK1R co-agonism somehow decreased neuronal activation (Figure 3), we focused our analysis on genes enriched in inhibited neurons. A total of 214 genes were differentially expressed in neurons regulated by the co-agonists (Supplementary Fig. 8 and Supplementary Table 7). Among these, 71 were differentially expressed selectively in neurons responding to the combination treatment but not the monotherapies, among which 26 were enriched in inhibited neurons (Figure 7A–B). A total of 13 genes were more enriched in neurons in response to the combination treatment vs the monotherapies (Figure 7C), but all of these were enriched in activated neurons (dER > 0). We determined the cellular distribution of all of these genes in the MBH using the published single-cell RNAseq database from the MBH [29] and found that none of them were labelled a discrete group of MBH cells, thus making it difficult to use these data to identify a marker for the discrete cell population inhibited by the treatment in the ARH. Nevertheless, *Adcyap1r1* was one of the top markers of neurons inhibited by the treatment (Supplementary Fig. 7d). *Adcyap1r1* encodes a receptor for the neurotransmitter PACAP. Using RNAscope, we determined the activation status of MBH *Adcyap1r1*^+^ neurons in response to the combinatorial treatment. Using this technique, we confirmed that GLP-1R and CCK1R co-agonism decreased neuronal activation in the ARH (Figure 7D,F) and that activity of ARH *Adcyap1r1*^+^ neurons significantly decreased (Figure 7E,F). These results confirmed that GLP-1R and CCK1R co-agonism inhibited ARH neurons and identified *Adcyap1r1* as a marker of the ARH neuronal population inhibited by the treatment.

## 4 Discussion

Combinatorial therapies are under intense investigation for developing more efficient weight-suppressing drugs to treat hyperphagic obesity; however, little is known about how these drug combinations act in the central nervous system to produce enhanced satiety and weight loss. In this study, we used a combination of approaches to decipher the behavioural, neurochemical, and molecular mechanisms engaged downstream from the combination of GLP-1R and CCK1R agonists that produced sustained appetite and weight suppression in lean and diet-induced obese mice. We found that sustained hyperphagia was essentially mediated by a decrease in meal frequency, supporting the idea that central mechanisms mediating satiety rather than satiation are engaged in the sustained appetite-suppressive effect of this combination. We found that only a few brain sites had increased neuronal activation in response to GLP-1R and CCK1R co-agonism, including the NTS in the caudomedial brainstem, which showed the most robust increased neuronal activation in response to GLP-1R and CCKR1 co-agonism. In the NTS, none of the candidate neuronal subpopulations that we investigated showed increased activation in response to the co-agonist treatment. However, PhosphoTRAP revealed that neurons expressing the CGRP receptor *Calcrl* were only activated in response to the combinatorial treatment, and a wide-spectrum CLR antagonist blunted the anorectic response to GLP-1R and CCK1R co-agonism. GLP-1R and CCK1R co-agonism also decreased the activation of ARH AGRP neurons, and PhosphoTRAP in the MBH samples indicated that neurons inhibited by the treatments expressed PACAP receptor. Collectively, these studies identified neuronal populations involved in the appetite- and weight-suppressive effect of GLP-1R and CCK1R co-agonism.

The analysis of the meal pattern responses to the agonists provided insights into the behavioural mechanisms engaged to produce sustained hypophagia and weight loss in response to GLP-1R and CCK1R co-agonism. AC3174 monotherapy only elicited minor effects on any of the measured parameters, whereas AC170222 produced a robust but transient hypophagic response. In the mice treated with both agonists, the early reduction in meal size was rapidly replaced by a sustained reduction in meal frequency, indicating that the subthreshold dose of GLP-1R agonist prevented the compensatory increase in meal frequency observed in the mice treated with CCK1R agonist alone. This produced a significant weight loss after 5 days. Whether the weight loss was solely due to a decrease in energy intake would warrant additional investigations, and a decrease in energy expenditure could have contributed to the weight loss as well as previously reported [10]. Importantly, the results from this behavioural analysis revealing a regulation of meal frequency rather than meal size to elicit the sustained response to GLP-1R and CCK1R co-agonism strongly suggested a role of forebrain appetite-regulating circuits in this response [30].

In line with the lack of feeding response to the GLP-1R agonist alone, neuronal activation following this treatment was mild across all of the brain sites that we examined. In particular, the GLP-1R agonist did not activate hindbrain GLP-1R^+^ neurons. In the ARH, a larger population of GLP-1R^+^ neurons (±60%) was activated in response to this treatment, but 40% of these neurons were already activated in response to vehicle treatment, supporting the conclusion that activation of GLP-1R^+^ neurons in the ARH was predominantly independent of GLP-1R agonism. Together with the observation that many GLP-1R^−^ neurons were activated in response to AC3174 in the ARH, we propose that fos activation in the ARH in response to AC3174 may not primarily be driven by GLP-1R signalling in activated neurons, but rather the product of the integration of afferent signals by many (GLP-1R^+^ and GLP-1R^−^) neurons. In contract, the robust anorectic response to the CCK1R agonist was accompanied by an increase in neuronal activation across many brain sites. Remarkably, co-agonism produced increased neuronal activation in only three brain sites, most significantly in the NTS, demonstrating this site's role as the main neuronal substrate engaged in the integration of GLP-1R and CCK1R signalling. Of note, the LPBN also showed increased activation, but c-fos-positive cells were located in the dorsal portion of the LPBN, a site distinct from the ventrolateral PBN, which contains CGRP neurons implicated in aversive responses [31].

The neurochemical characterisation of the activated neurons in response to the three treatments revealed that the population of neurons activated by the co-agonism was distinct from primary sensing neurons expressing CCK1R and GLP-1R, which indicated that the integration of these signals occurred at the level of a downstream integration point still within the DVC. This characterisation also increased our understanding of the regulation of MBH neuronal populations in response to the drugs and revealed that the CCK1R agonist and combinatorial treatment activated POMC neurons and inhibited AGRP neurons, with a higher effect of the combinatorial treatment, suggesting that these responses may have been involved in the increased anorectic response to GLP-1R and CCK1R co-agonism. However, in the DVC, we failed to identify the neuronal subpopulation specifically activated by the combination using this approach and excluded a contribution of DVC TH, GLP1R, and POMC neurons in this effect.

Using PhosphoTRAP as a hypothesis-generating tool to identify molecular markers for neurons regulated in the DVC and MBH specifically in response to the combination treatments, we identified *Calcrl* as a marker of DVC neurons activated by GLP-1R and CCK1R co-agonism. This is a druggable target, making these findings potentially translatable for developing new anti-obesity drugs. However, further research is required to determine the extent to which *Calcrl* signalling mediates the anorexigenic and weight-reducing effects of GLP-1/CCK co-administration. Of note, although PhosphoTRAP captured the majority of activated neurons in the MBH and DVC, this technique was not as successful in the hindbrain, with a lower enrichment in activity-dependent genes in the ip samples. This may have been because this region had virtually no activated cells at baseline and in the control group. This approach used in the MBH revealed that the GLP-1R/CCK1R combination inhibited neurons expressing PACAP receptors. Whether PACAP receptor signalling is engaged in the response remains to be established. Central PACAP signalling has previously been shown to suppress food intake [32] mostly via VMH PACAP signalling [33,34]. However, recent data demonstrated that PVH PACAP input to the ARH produced hyperphagia and that PACAP receptor signalling activated AGRP neurons [35]. In this study, the combinatorial treatment did not modify the activity of VMH PACAP receptor neurons but inhibited ARH PACAP receptor neurons. Together with the reduced activity of ARH AGRP neurons in response to this treatment, these results suggested that GLP-1R and CCK1R co-agonism reduced the excitatory PVH to AGRP orexigenic drive.

In conclusion, these studies revealed behavioural, neurochemical, and molecular mechanisms involved in the integration of two gut-derived peptides that produced enhanced appetite- and weight-suppressing effects and demonstrated the value of a novel approach to identify central druggable targets involved in the integration of combinatorial therapies.

## Author contributions

ER, SB, SW, IP, BQ, MM, NH, TD, YS, FG, FR, IP, and CB conducted the experiments. ER, BQ, BL, and CB performed data analysis. CB, GY, DB, and JT designed the experiments. CB, ER, GY, DB, and JT wrote the manuscript.

## Appendix A Supplementary data

Supplementary Tables 1 **Supplementary Tables 1:** Qualitative analysis of c-fos expression throughout the neuraxis in response to the 4 treatments. Green: Brain areas with more activation in response to AC3174. Purple: Brain areas with more activation in response to AC170222. Yellow: Brain areas with more activation in response to AC170222 + Ac3174. Supplementary Tables 1

Supplementary Figures **Supplementary Figure 1:** Feeding response to increasing doses of AC3174 (a-e) and CCK-8 (c and d) in the mice fed a chow (a and c) or high-fat diet (d and e) for 3 weeks. Body weight of the mice fed a low- or high-fat diet for 3 weeks (b). Data are means ± sem. ∗*p* < 0.05 and ∗∗*p* < 0.01. The mice were 9 weeks old at the beginning of the studies (±26 g). **Supplementary Figure 2:** AC3174 and AC170222 did not produce pica behaviour in the rats. Two h (a) and 24 h (b) chow intake, body weight change (c), 6 h (d) and 24 h (e) kaolin intake in the rats following the injection of saline, cisplatin, GLP-1R agonist AC3174, CCK1R agonist AC170222, or AC3174 + AC170222. Data are means ± sem. ∗*p* < 0.05, ∗∗*p* < 0.01, ∗∗∗*p* < 0.001, and ∗∗∗∗*p* < 0.0001. **Supplementary Figure 3:** Average meal size (a) and meal number (b) over the first 12 h, average meal size (c) and meal number (d) over the 5 days of treatment with saline, GLP-1R agonist AC3174 (3 μg/kg), CCK1R agonist AC170222 (30 μg/kg), or AC3174 + AC170222. Data are means ± sem. ∗*p* < 0.05, ∗∗*p* < 0.01, and ∗∗∗*p* < 0.001 vs saline. Mice were 9 weeks old at the beginning of the studies (±26 g). **Supplementary Figure 4:** Neuronal activation in response to acute GLP-1R and CCK1R co-agonism in the lateral parabrachial nucleus (LPBN) and the central amygdala (CeA). Representative images showing c-fos immunolabelling in the LPBN and CeA 80 min after an i.p. administration of saline, GLP-1R agonist AC3174, CCK1R agonist AC170222, or AC3174 + AC170222. The mice were 9 weeks old at the beginning of the studies (±26 g). **Supplementary Figure 5:** Expression of phosphorylated protein S6 in activated neurons. Quantification and representative images showing the co-localisation of c-fos and pS6 in the ARH, AP, and NTS in response to i.p. administration of saline, GLP-1R agonist AC3174, CCK1R agonist AC170222, or AC3174 + AC170222. Data are means ± sem. **Supplementary Figure 6:** Neuroanatomical-specific tissue microdissection in the PhosphoTRAP assay and enrichment in activity-dependent genes in the ip samples. We used the Allen Atlas to identify region-specific genes in the ARH (a: *Pomc*, *Agrp*, *Cartpt*, *Mr5a1*, and *Kiss1*) and DVC (b: *Th*, *Dbh*, *Pomc*, *Nnat*, *GFRAL*, and *Gcg*). We also confirmed the absence of PVH-specific genes (a: *Sim1*, *Trh*, and *Crh*). MBH (c) and DVC (d) samples were enriched in activity-dependent genes. **Supplementary Figure 7:** PhosphoTRAP assay identified the molecular profile and neurons activated or inhibited by the mono-agonist and dual agonist treatments in the DVC. (a) Number of genes significantly enriched in activated or inhibited neurons in response to each treatment. (b) Genes of interest (secreted and plasma membrane proteins) enriched in neurons responding to each treatment. Enrichment ratios of transcripts enriched in neurons responding to all of the treatments (c) to AC3174 and the combo (d) and AC170222 and the combo (e). Data are means ± sem. Means sharing a letter are not statistically different. All of the genes encoding secreted or plasma membrane protein are squared in red. **Supplementary Figure 8:** PhosphoTRAP assay identified the molecular profile and neurons activated or inhibited by the mono-agonist and dual agonist treatments in the MBH. (a) Number of genes significantly enriched in activated or inhibited neurons in response to each treatment. (b) Genes of interest (secreted and plasma membrane proteins) enriched in neurons responding to each treatment. Enrichment ratios of transcripts enriched in neurons responding to all of the treatments (c) to AC3174 and the combo (d) and AC170222 and the combo (e). Data are means ± sem. Means sharing a letter are not statistically different. All of the genes encoding secreted or plasma membrane protein are squared in red. Supplementary Figures
