# Supplementary material for: Behavioural and neurochemical mechanisms underpinning the feeding-suppressive effect of GLP-1/CCK combinatorial therapy
Source: Mol Metab. 2020 Nov 19;43:101118. doi: 10.1016/j.molmet.2020.101118 (PMC7720077; doi:10.1016/j.molmet.2020.101118)
Supplement: Supplementary Figures — Supplementary Figure 1: Feeding response to increasing doses of AC3174 (a-e) and CCK-8 (c and d) in the mice fed a chow (a and c) or high-fat diet (d and e) for 3 weeks. Body weight of the mice fed a low- or high-fat diet for 3 weeks (b). Data are means ± sem. ∗p < 0.05 and ∗∗p < 0.01. The mice were 9 weeks old at the beginning of the studies (±26 g). Supplementary Figure 2: AC3174 and AC170222 did not produce pica behaviour in the rats. Two h (a) and 24 h (b) chow intake, body weight change (c), 6 h (d) and 24 h (e) kaolin intake in the rats following the injection of saline, cisplatin, GLP-1R agonist AC3174, CCK1R agonist AC170222, or AC3174 + AC170222. Data are means ± sem. ∗p < 0.05, ∗∗p < 0.01, ∗∗∗p < 0.001, and ∗∗∗∗p < 0.0001. Supplementary Figure 3: Average meal size (a) and meal number (b) over the first 12 h, average meal size (c) and meal number (d) over the 5 days of treatment with saline, GLP-1R agonist AC3174 (3 μg/kg), CCK1R agonist AC170222 (30 μg/kg), or AC3174 + AC170222. Data are means ± sem. ∗p < 0.05, ∗∗p < 0.01, and ∗∗∗p < 0.001 vs saline. Mice were 9 weeks old at the beginning of the studies (±26 g). Supplementary Figure 4: Neuronal activation in response to acute GLP-1R and CCK1R co-agonism in the lateral parabrachial nucleus (LPBN) and the central amygdala (CeA). Representative images showing c-fos immunolabelling in the LPBN and CeA 80 min after an i.p. administration of saline, GLP-1R agonist AC3174, CCK1R agonist AC170222, or AC3174 + AC170222. The mice were 9 weeks old at the beginning of the studies (±26 g). Supplementary Figure 5: Expression of phosphorylated protein S6 in activated neurons. Quantification and representative images showing the co-localisation of c-fos and pS6 in the ARH, AP, and NTS in response to i.p. administration of saline, GLP-1R agonist AC3174, CCK1R agonist AC170222, or AC3174 + AC170222. Data are means ± sem. Supplementary Figure 6: Neuroanatomical-specific tissue microdissection in the PhosphoTRAP assay and enr [file mmc2.docx]

Suppl. Fig 1: Feeding response to increasing doses of AC3174 (a-e) and CCK-8 (c, d) in mice fed a chow (a,c) or high-fat (d,e) diet for 3 weeks. Body weight in mice fed a low or high fat diet for 3 weeks (b). Data are means ± sem. *: p<0.05; **: p<0.01. Mice were 9-wks old at the beginning of the studies (±26g).

Supplementary Figure 2: AC3174 and AC170222 do not produce pica behavior in rats. 2h (a) and 24h (b) chow intake, body weight change (c), 6h (d) and 24h (3) kaolin intake in rats following the injection of saline, cisplatin, GLP-1R agonist AC3174, CCK1R agonist AC170222 or AC3174+AC170222. Data are means ± sem. *: p<0.05; **: p<0.01, ***: p<0.001, ****: p<0.0001.


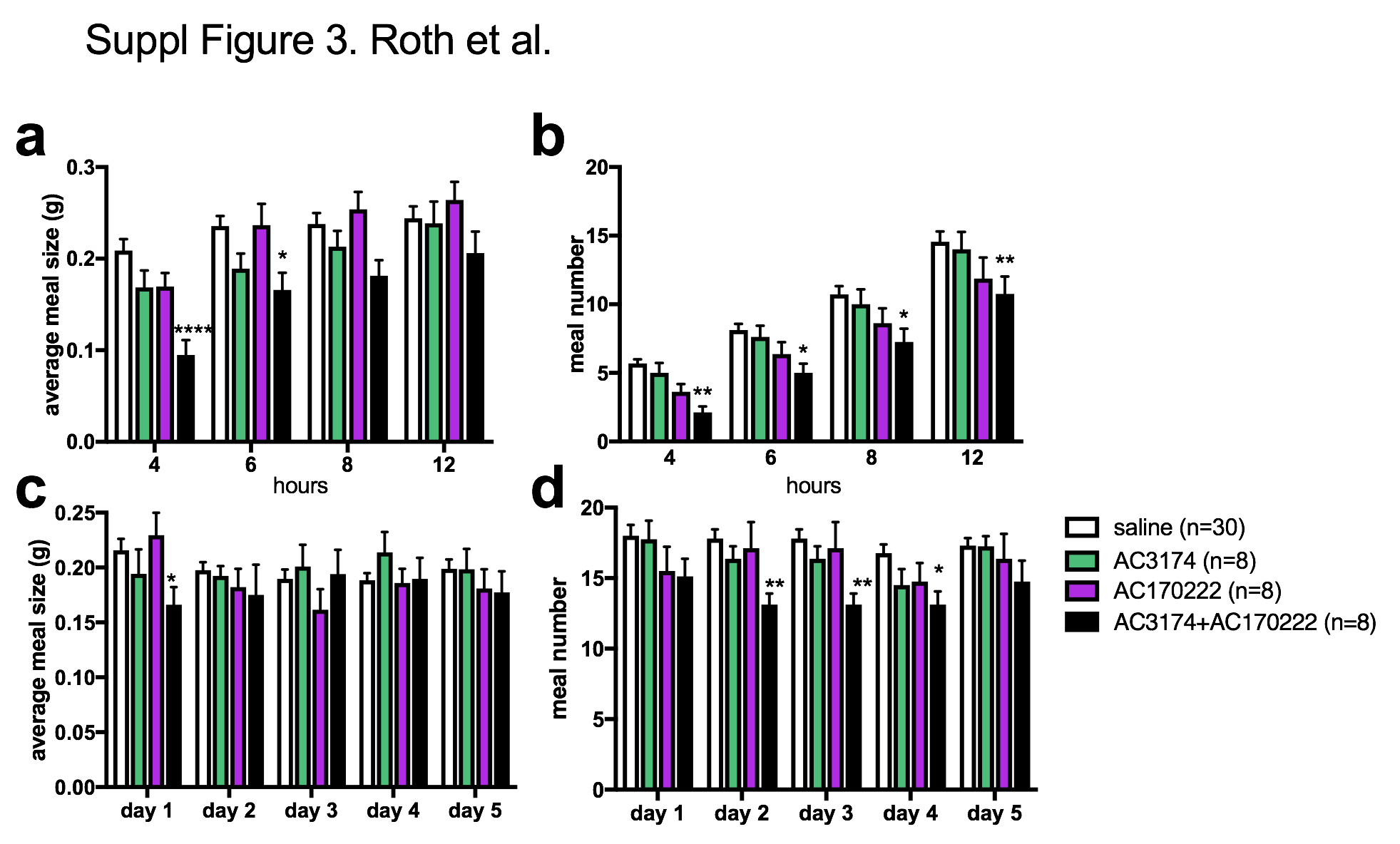


Suppl. Figure 3: Average meal size (a) and meal number (b) over the first 12 h, average meal size (c) and meal number (d) over the 5 days treatment with saline, GLP-1R agonist AC3174 (3μg/kg), CCK1R agonist AC170222 (30μg/kg) or AC3174+AC170222. Data are means ± sem. *: p<0.05; **: p<0.01, ***: p<0.001 vs. saline. Mice were 9-wks old at the beginning of the studies (±26g).

Suppl. Fig 4: Neuronal activation in response to acute GLP-1R and CCK1R co-agonism in the lateral parabrachial nucleus (LPBN) and the Central Amygdala (CeA). Representative images showing c-fos immunolabelling in the LPBN and CeA 80 min after an ip. administration of saline, GLP-1R agonist AC3174, CCK1R agonist AC170222 or AC3174+AC170222. Mice were 9-wks old at the beginning of the studies (±26g).


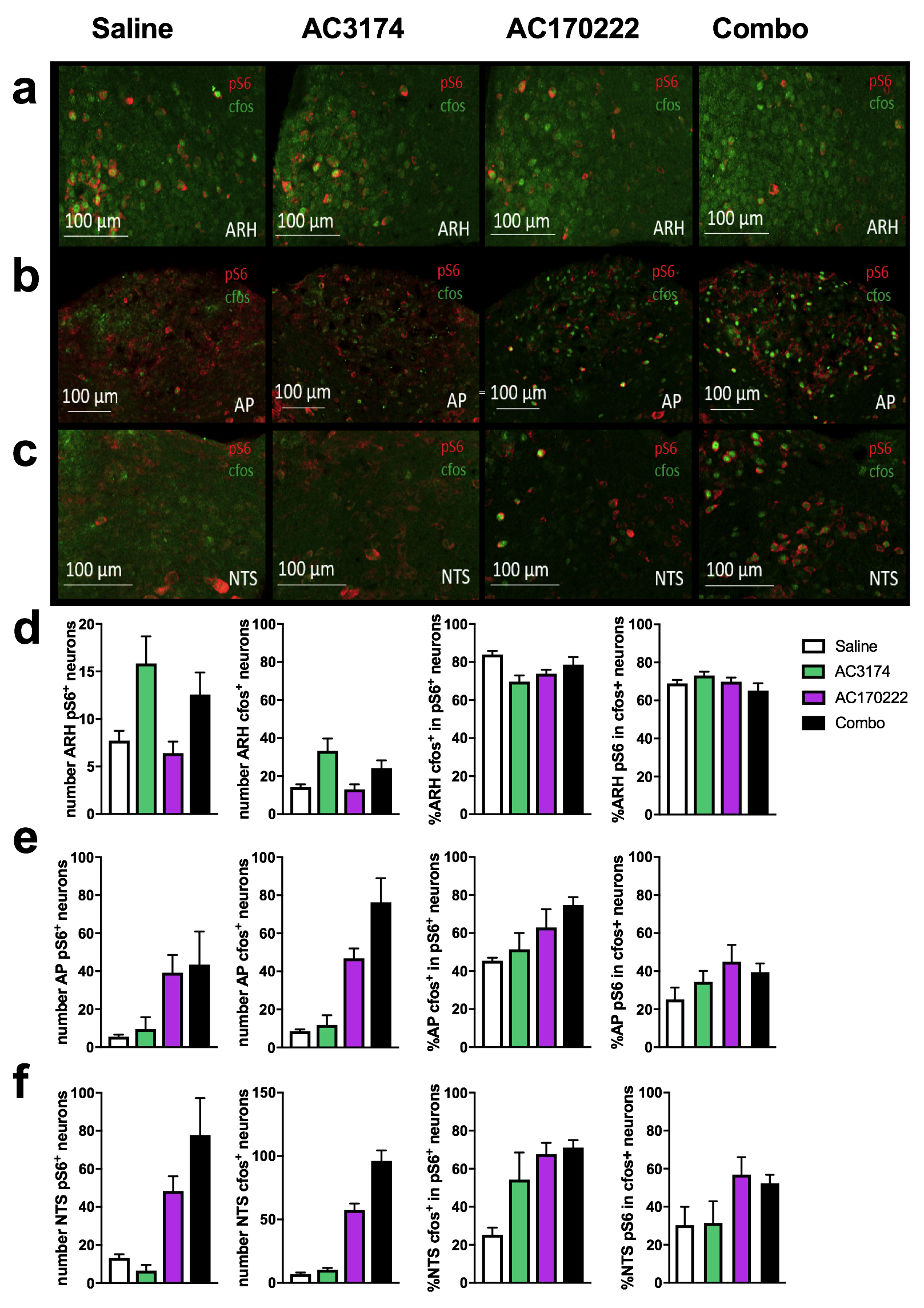


**Suppl. Fig 5: Expression of phosphorylated protein S6 in activated neurons**. Quantification and representative images showing the colocalization of c-fos and p-S6 in the ARH, AP and NTS in response to ip administration of saline, GLP-1R agonist AC3174, CCK1R agonist AC170222 or AC3174+AC170222. Data are means ± sem.


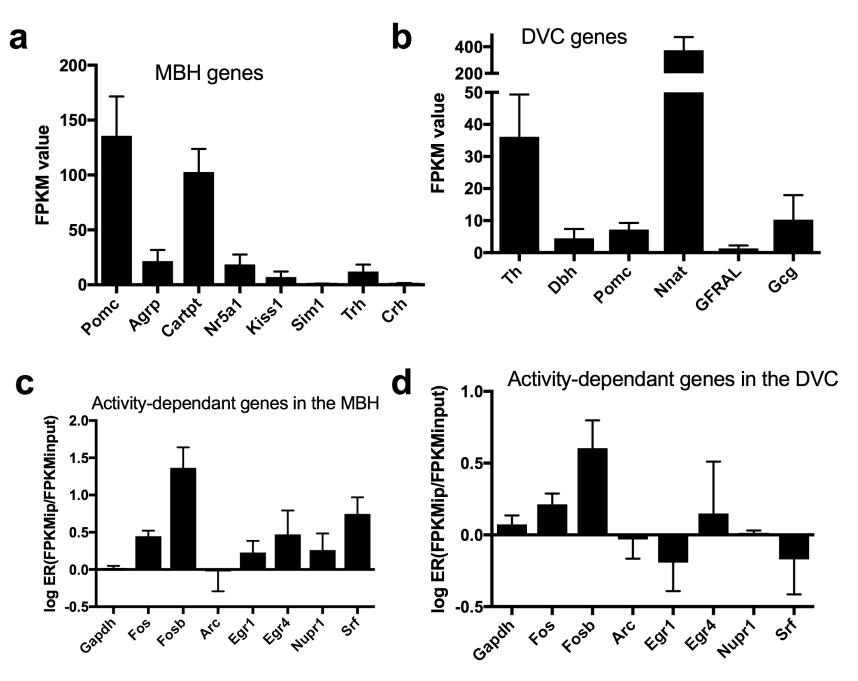


**Suppl. Fig 6: Neuroanatomical specific of tissue microdissection in the Phospho-TRAP assay and enrichment in activity-dependent genes in the ip samples .**  We used the Allen Atlas to identify region specific genes in the ARH (a, *Pomc, Agrp, Cartpt, Mr5a1, Kiss1*) and DVC (b, *Th, Dbh, Pomc, Nnat, GFRAL, Gcg*). We also confirmed the absence of PVH specific genes (a, *Sim1, Trh, Crh*). MBH (c) and DVC (d) samples are enriched in activity-dependent genes.


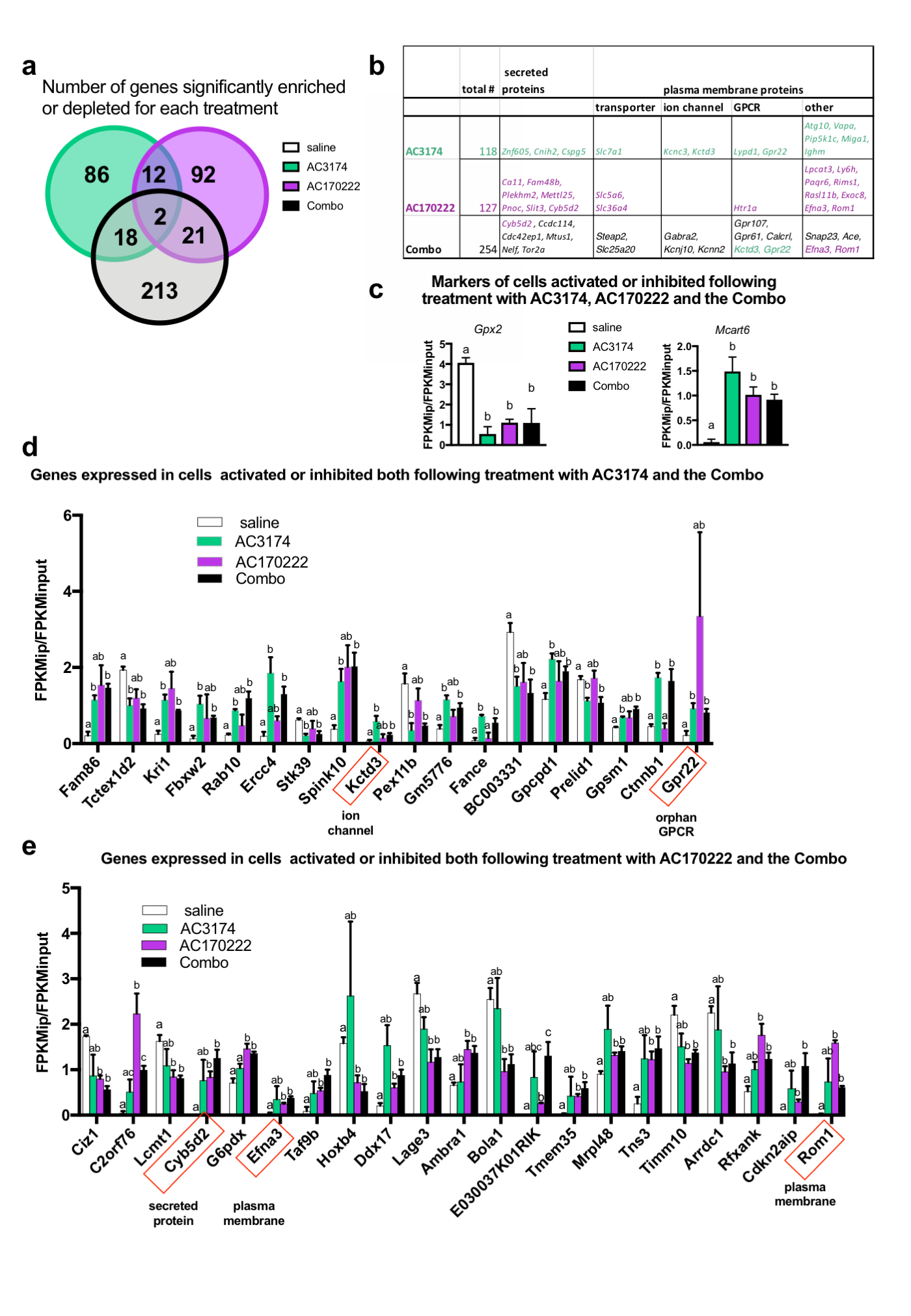


**Suppl. Fig. 7: PhosphoTRAP assay identifies the molecular profile and neurons activated or inhibited by the mono and dual agonist treatments in the DVC.** (a) Number of genes significantly enriched in activated or inhibited neurons in response to each treatment. (b) Genes of interest (secreted and plasma membrane proteins) enriched in neurons responding to each treatment. Enrichment ratios of transcripts enriched in neurons responding to all treatments (c), to AC3174 and the combo (d) and to AC170222 and the combo (e). Data are means ± sem. Means sharing a letter are not statistically different. All genes encoding secreted or plasma membrane protein are squared in red.

**Suppl. Fig. 8: PhosphoTRAP assay identifies the molecular profile and neurons activated or inhibited by the mono and dual agonist treatments in the MBH.** (a) Number of genes significantly enriched in activated or inhibited neurons in response to each treatment. (b) Genes of interest (secreted and plasma membrane proteins) enriched in neurons responding to each treatment. Enrichment ratios of transcripts enriched in neurons responding to all treatments (c), to AC3174 and the combo (d) and to AC170222 and the combo (e). Data are means ± sem. Means sharing a letter are not statistically different. All genes encoding secreted or plasma membrane protein are squared in red.
